# A Chatbot to Engage Parents of Preterm and Term Infants on Parental Stress, Parental Sleep, and Infant Feeding: Usability and Feasibility Study

**DOI:** 10.2196/30169

**Published:** 2021-10-26

**Authors:** Jill Wong, Agathe C Foussat, Steven Ting, Enzo Acerbi, Ruurd M van Elburg, Chua Mei Chien

**Affiliations:** 1 Precision Nutrition D-lab Danone Nutricia Research Singapore Singapore; 2 Cytel Singapore Private Limited Singapore Singapore; 3 NLYTICS Pte. Ltd. Singapore Singapore; 4 Department of Pediatrics Emma Children’s Hospital Amsterdam University Medical Center Amsterdam Netherlands; 5 Nutrition4Health Hilversum Netherlands; 6 Department of Neonatology KK Women’s and Children’s Hospital Singapore Singapore; 7 Duke-NUS Graduate School of Medicine Singapore Singapore; 8 Yong Loo Lin School of Medicine National University of Singapore Singapore Singapore; 9 Lee Kong Chian School of Medicine Nanyang Technological University Singapore Singapore

**Keywords:** chatbot, parental stress, parental sleep, infant feeding, preterm infants, term infants, sleep, stress, eHealth, support, anxiety, usability

## Abstract

**Background:**

Parents commonly experience anxiety, worry, and psychological distress in caring for newborn infants, particularly those born preterm. Web-based therapist services may offer greater accessibility and timely psychological support for parents but are nevertheless labor intensive due to their interactive nature. Chatbots that simulate humanlike conversations show promise for such interactive applications.

**Objective:**

The aim of this study is to explore the usability and feasibility of chatbot technology for gathering real-life conversation data on stress, sleep, and infant feeding from parents with newborn infants and to investigate differences between the experiences of parents with preterm and term infants.

**Methods:**

Parents aged ≥21 years with infants aged ≤6 months were enrolled from November 2018 to March 2019. Three chatbot scripts (stress, sleep, feeding) were developed to capture conversations with parents via their mobile devices. Parents completed a chatbot usability questionnaire upon study completion. Responses to closed-ended questions and manually coded open-ended responses were summarized descriptively. Open-ended responses were analyzed using the latent Dirichlet allocation method to uncover semantic topics.

**Results:**

Of 45 enrolled participants (20 preterm, 25 term), 26 completed the study. Parents rated the chatbot as “easy” to use (mean 4.08, SD 0.74; 1=very difficult, 5=very easy) and were “satisfied” (mean 3.81, SD 0.90; 1=very dissatisfied, 5 very satisfied). Of 45 enrolled parents, those with preterm infants reported emotional stress more frequently than did parents of term infants (33 vs 24 occasions). Parents generally reported satisfactory sleep quality. The preterm group reported feeding problems more frequently than did the term group (8 vs 2 occasions). In stress domain conversations, topics linked to “discomfort” and “tiredness” were more prevalent in preterm group conversations, whereas the topic of “positive feelings” occurred more frequently in the term group conversations. Interestingly, feeding-related topics dominated the content of sleep domain conversations, suggesting that frequent or irregular feeding may affect parents’ ability to get adequate sleep or rest.

**Conclusions:**

The chatbot was successfully used to collect real-time conversation data on stress, sleep, and infant feeding from a group of 45 parents. In their chatbot conversations, term group parents frequently expressed positive emotions, whereas preterm group parents frequently expressed physical discomfort and tiredness, as well as emotional stress. Overall, parents who completed the study gave positive feedback on their user experience with the chatbot as a tool to express their thoughts and concerns.

**Trial Registration:**

ClinicalTrials.gov NCT03630679; https://clinicaltrials.gov/ct2/show/NCT03630679

## Introduction

Caring for infants can lead to parental anxiety and psychological distress especially for first-time parents and particularly within the first 6 months after birth [1]. Multiple studies have demonstrated that parental stress, anxiety, and psychological distress are not only short-term problems but may also have long-lasting effects on the child’s emotional, behavioral, and cognitive development [1]. These are more prominent for parents of preterm infants than for parents of term infants [2]. An assessment of maternal psychological distress in singleton versus multiple-birth preterm infants found that mothers with multiple births had greater posttraumatic stress symptoms, anxiety at discharge, and depressive symptoms at 6 months as compared to mothers of singletons [3]. In a follow-up clinic evaluation of parents and their preterm infants, many reported parental concerns about medical and developmental outcomes that were unsupported by their child’s diagnosis [4]. Among mothers of school-aged children who were born late preterm and admitted to an intensive care unit (ICU), there was a significant 18-fold increase in total stress compared to stress among mothers of term children [5]. In a parallel study group involving mothers of school-aged children who were born late preterm but not admitted to the ICU, there was also a 24-fold increase in total stress when compared to the mothers of term-born children [5].

Besides experiencing initial stress directly after birth, parents need to adapt to the new situation after hospital discharge (or at home) and develop confidence in caring for their newborns themselves. These adjustments and care transition from a medical facility to home may be associated with increased stress and loss of sleep. Although sleep disturbance is most commonly associated with the early postpartum period, parents may continue to experience disturbed sleep for some months after birth [6,7]. In addition to sleep disturbance, infant feeding, including the frequency and type of nutrition, is another potential stressor and is associated with depressive symptoms and higher stress ratings [8]. Parents of preterm infants often face issues with frequency of feeding, and their infants also often start solid food later in life [9]. Relatively little knowledge is available on the parental experience in the areas of stress, sleep, and infant feeding during this period of change in family life.

Web-based interventions for mental health have shown some success in conditions such as depression and anxiety [10]. The remote presence of human support through these interventions has been shown to outperform self-guided interventions and achieve higher rates of participant adherence [10]. Studies have shown that these positive outcomes were achieved by implementing periodic prompts and frequent interactions with participants [11]. However, such interactive features are highly therapist intensive. Chatbot apps which can simulate humanlike conversations [12] have become popular in recent years. These kind of chatbot apps can provide computer-generated responses to a user in real time, mimicking conversational interactions with another human via instant electronic messaging [13]. This technology, coupled with use of mobile devices, presents possibilities to collect data in real time while reducing the workload of the therapist. Although chatbots are used in many apps, one of the more innovative areas of development is for interactive data collection in the health care sector [14].

In this study, chatbot technology was used to provide an interactive conversation platform to engage parents of newborn infants who were recently discharged from hospital in the areas of parental stress and sleep, and infant feeding. To our knowledge, there have been no studies published on the use of a chatbot as an interactive conversational tool for parents to provide information in these subject areas. The objective of this study is to explore the feasibility and usability of chatbot technology to gather real-life, in-home conversation data on 3 domains (parental stress, sleep, and infant feeding) from parents with newborn infants and investigate the differences between parents of preterm and term infants in these 3 domains using these conversation data.

## Methods

This observational study was conducted from November 2018 to March 2019. Participants were recruited from a tertiary referral maternity hospital in Singapore. The study was approved by the SingHealth Centralised Institutional Review Board, Singapore, and registered at ClinicalTrials.gov (NCT03630679).

### Study Population

The study population comprised parents aged ≥21 years with healthy infants who were ≤6 months of age and had been discharged from the hospital at the time of enrollment. Eligible parents had to be proficient in the English language, have in-home access to a reliable internet connection, own a tablet or a mobile device suitable for electronic communication and assessment, and be able to comply with the required study tasks. Nonsingleton infants or those known to have current or previous illnesses or conditions which might interfere with the study outcome or who were participating in any other clinical studies were excluded. Parents with a past or present history of mental illness, single parents, or parents who had any acute or chronic illnesses or who were assessed by the investigators to be unable or unwilling to comply with the study protocol requirements were excluded. Written informed consent was obtained from all eligible parents.

### Study Design

Participants for this observational study were screened based on the above inclusion and exclusion criteria. After providing informed consent, eligible participants were given access to download the ClaimIt app (ObvioHealth), which provided access to electronic questionnaires (eQuestionnaires) and the study chatbot, on to their mobile devices. ClaimIt is a commercially available mobile app for data collection in virtual or hybrid research studies that require no or minimal use of physical study sites. Participants completed an electronic Screening eQuestionnaire in ClaimIt to confirm their eligibility for enrollment. The study population included 2 groups: “preterm” (parents of preterm infants at gestational age <37 weeks) and “term” (parents of term infants at gestational age ≥37 weeks).

### Collection of Conversation Data, Ease of Use, and Satisfaction Ratings

ClaimIt was made available to participants so they could perform specific study-related tasks and receive study information. The participants were given instructions via the ClaimIt app on how to use the chatbot and were asked to interact with the chatbot at least 3 times a week over a maximum 28-day period. The chatbot is an interactive conversational app that was built as a component of the ClaimIt app specifically for this study. The chatbot conversed with users through an online platform. The chatbot was programmed using scripts to respond appropriately whenever a user initiated a conversation. The chatbot scripts included open-ended and closed-ended (multiple-choice) questions and responses. There were 3 conversation scripts, 1 for each of the 3 domains of interest, which included stress, sleep, and feeding (Multimedia Appendix 1).

Participants also received notifications on the first day of each week to remind them to complete the required number of interactions with the chatbot at their convenience. Reminder notifications were triggered on the first day of each week for the participant to complete 3 interactions over the week. Study compliance was monitored by the study team and principal investigator, and contact with the participants was made electronically, and if needed, by telephone. All study data were collected via the ClaimIt app running on participants’ mobile devices. Transcripts of the chatbot conversations were accessed and reviewed by the study team.

Each participant completed the Usability eQuestionnaire in the ClaimIt app at the end of the study (Multimedia Appendix 2). The questionnaire comprised 16 questions, including closed-ended (binary or 5-point Likert scale) and open-ended responses. Participants were asked to rate ease of use and satisfaction separately for the ClaimIt and chatbot components.

### Conversation Data Processing and Descriptive Statistical Analysis of Closed-Ended Questions

A sample size of 40 participants was planned to permit reporting of descriptive summary statistics for the categorical and quantitative response data collected using the chatbot. The expected dropout rate was 25%. If this threshold was exceeded despite the investigators’ efforts to contact participants who were lost to follow-up, a maximum of 10 additional participants could be enrolled to replace the participants who dropped out. Completed chatbot interactions from participants who dropped out were included in the conversation analysis.

**Figure 1:**
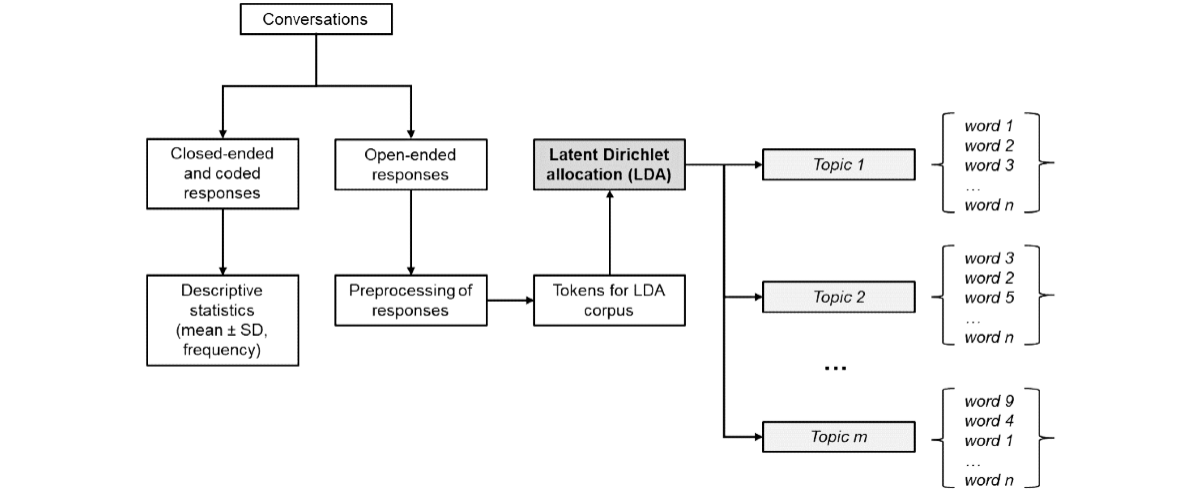
Workflow for conversation data processing and semantic analysis by latent Dirichlet allocation (LDA) topic modeling.

Each raw chatbot conversation was processed by separating open-ended responses from responses to closed-ended questions and suitably coded open-ended questions (Figure 1). Descriptive statistics were used to summarize the responses for closed-ended and coded open-ended responses from the Usability eQuestionnaire and chatbot conversations. Continuous data are presented using mean and SD or range, and categorical responses are presented using frequency and percentage. Descriptive summaries are also presented by group (preterm and term). No significance testing was performed. Statistical analyses were performed using SAS 9.4.

### Semantic Analysis of Chatbot Conversations

We used the latent Dirichlet allocation (LDA) [15] method to model and extract knowledge about semantic topics within our corpus (body of text), which was derived from open-ended responses within the chatbot conversation data. In the context of LDA, each conversation is represented as a mixture of topics, and each topic is associated with a collection of words. Each word is represented as belonging to a topic with a certain probability, and different words in a conversation may belong to different topics. The objective is to find a set of representative words for each topic. In LDA-based topic modeling, the actual semantic meaning of each topic cannot be automatically inferred from the data. Instead, the link between a topic and its semantic meaning (a concept that a human would understand) has to be made by a person based on subjective judgement. A valid topic model, however, makes linking a semantic meaning to a topic a trivial task; for example, the words “beach,” “sand,” “sun,” and “relax” once grouped together by the LDA algorithm, would be easily recognized by most persons as a concept for “holiday.”

Besides LDA, other natural language processing methodologies that have been explored for topic modeling include latent semantic analysis/indexing (LSA/LSI) [16], probabilistic latent semantic analysis (pLSA) [17], and nonnegative matrix factorization (NMF) [18]. In general, these methods infer topics from document-level word co-occurrences by modeling each document as a mixture of topics. However, such inference is limited by the sparsity of word co-occurrence patterns when learning from short texts, for example, those on social network platforms. Other issues encountered with short texts include slang, spelling or grammatical errors, and nonmeaningful or noisy words.

LSA/LSI is nonprobabilistic and relies on a mathematical procedure, known as singular value decomposition [19], and can make use of a term frequency-inverse document frequency matrix which assigns large weights to terms that occur frequently within a document but rarely within the corpus, and vice-versa. As LSA/LSI techniques typically require a large corpus in order to produce accurate groupings or topic models, they were not considered an appropriate methodology for this study. Another approach, pLSA, replaces the singular value decomposition procedure with a probabilistic one. Although pLSA represents a valid alternative to LDA, overfitting is known to be less controllable when using pLSA in its basic form [20]. NMF uses a matrix factorization method to simultaneously perform dimension reduction on a term-document matrix and clustering of terms to extract topics [21].

Albalawi et al [22] evaluated a number of topic modelling methods for short texts and concluded that LDA and NMF provided the best learned descriptive topics and addressed the limitations affecting the other topic modeling methods. Compared with NMF, LDA has produced more consistent results [22] and has been applied to studies in various domains with a number of toolkits readily available for its implementation. Based on these considerations, LDA was deemed the most suitable method for analyzing conversation data from the chatbot.

Chatbot conversations were analyzed independently for the stress, sleep, and feeding domains. As with online apps, the user-generated texts in this study were often limited in length. Therefore, the average conversation length was increased by merging multiple conversations collected from the same participant over the study period into a single conversation for each domain (Multimedia Appendix 3). These merged conversations were then used for LDA topic modeling.

#### Preprocessing of Open-Ended Responses

The first preprocessing step was to eliminate stop words (ie, those that do not carry information about topics). Stop words for the English language [23] were removed as were additional stop words identified as being specific to each of the 3 domains under consideration. We converted composite words into single words; for example, “not well” was converted to “not_well.” Local terms (“want,” “know,” “need,” “twice,” “not_well,” “well,” “need,” “went,” “couldn,” “occasion,” “not,” “babi,” “feel,” “okai,” “carri,” “unab,” “veri,” “left,” “right,” “care,” “affect,” “manag,” “everi,” “felt,” “time,” “sometim,” “sure,” “onli,” and “usual”) were also added to the stop word list.

Stemming of the remaining words in conversations was performed using the Gensim library [24]. Only stem tokens with a length greater than 3 letters were retained; shorter tokens were discarded. Tokens that appeared in fewer than 2 conversations in a single domain were also discarded as were tokens that appeared in more than 50% of the conversations of the domain. For conversations belonging to the feeding domain, product names and brand names were also removed. The resulting tokens formed the conversation corpus for the knowledge extraction to be performed by LDA-based topic modeling (Gensim 3.7.1 implementation [24]). The preprocessing workflow to derive the corpus for LDA machine learning is shown in Multimedia Appendix 3. The aim was to obtain a reduced set of words (corpus) for consideration when the LDA was used to extract topics from the chatbot conversations.

#### Knowledge Extraction

For each domain, 8 modeling sessions were performed, with the number of latent topics to be extracted set to a value from 2 through 9. Thus, a model was created for each setting (2 through 9 latent topics extracted). To obtain each model, we performed 10 learning runs by randomly changing the value of the random seed used to initialize the LDA procedure (ie, allocating a word to a topic), while the number of training passes (to determine the probability of the word belonging to a topic) was set at 100 for all runs. The best models for each domain could not be unequivocally identified based on a perplexity measure [25], and therefore human interpretation by domain experts was used. Human experts identified models with 3 or 4 topics as the most interpretable ones. Topics were visualized using LDAvis [26].

As a topic is a probability distribution over the entire dictionary of the corpus, only words with the highest probability values were deemed to be representative of the semantic meaning for that topic. We chose the 3 highest probability words within a topic to be most representative of the semantic concept associated with that topic. In simpler terms, one can think of these 3 highest probability words as the most frequently used words within that topic.

## Results

### Participants

**Figure 2:**
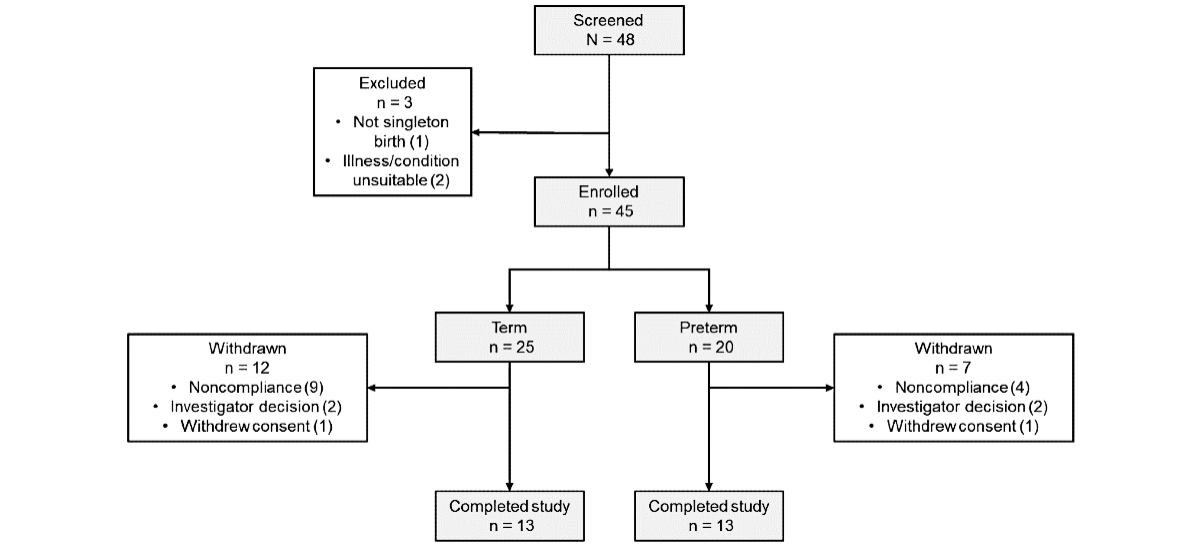
Participant flowchart.

A total of 48 parents were screened. Of these, 45 participants were enrolled in the study. This included 5 participants with term infants who were recruited to replace participants withdrawn from the study due to noncompliance. There were 45 infants (23 females, 51%; 20 preterm and 25 term infants). In all, 19 participants withdrew from the study: 13 (68%) participants failed to complete at least 5 interactions, 4 (21%) were withdrawn at the investigator’s decision, and 2 (11%) withdrew consent. A total of 26 participants, 13 in each group, completed the study (Figure 2).

All parents (n=45) were female. The mean age of the participants was 31.7 (SD 4.3) years while their infants were a mean 1.1 (SD 1.3) months old (Table 1). Participants completed 256 interactions with the chatbot, which included 259, 257, and 267 conversations on stress, sleep, and feeding, respectively.

**Table 1:** Characteristics of participants and chatbot responses.

Characteristic			Term (N=25)		Preterm (N=20)		Total (N=45)
Female gender (parent), n (%)			25 (100)		20 (100)		45 (100)
Female gender (infant), n (%)			13 (52)		10 (50.0)		23 (51)
Age of parents (years), mean (SD)			31.1 (4)		32.5 (5.0)		31.7 (4)
Age of infants (months), mean (SD)			1.1 (1)		1.2 (1)		1.1 (1)
**Completed conversations, n**							
	Stress domain	126		133		259	
	Sleep domain	125		132		257	
	Feeding domain	130		137		267	
Interactions (all 3 domains), n			125		131		256
**Merged conversations for LDA^a^ topic modeling^b^, n**							
	Stress domain	17		22		39	
	Sleep domain	17		22		39	
	Feeding domain	18		22		40	

^a^LDA: latent Dirichlet allocation.

^b^Within each of the 3 domains, conversations belonging to the same participant were merged into a single conversation. Completed chatbot interactions from participants who dropped out were included in the conversation analysis.

### Ease of Use and Satisfaction With the Chatbot

Of the 45 parents enrolled, 26 completed the study and the usability eQuestionnaire. Responses from these 26 participants (on a 5-point Likert scale; 1=very difficult, 5=very easy) showed that the chatbot was rated as “easy” to use (mean 4.08, SD 0.74). Preterm and term group parents who completed the study rated it similarly (preterm: mean 3.9, SD 0.86; term: mean 4.2, SD 0.60). The ClaimIt app was also perceived as “easy” to use (mean 4.19, SD 0.85) by both the preterm and term group parents (preterm: mean 4.0, SD 1.0; term: mean 4.38, SD 0.65).

Parents were “satisfied” with the chatbot (mean 3.81, SD 0.90; 1=very dissatisfied, 5=very satisfied) and also with the ClaimIt app (mean 3.81, SD 0.80). Participants in the preterm group registered between “neutral” and “satisfied” with the chatbot (mean 3.62, SD 0.96) and ClaimIt (mean 3.69, SD 0.85) app. Higher mean scores were observed in the term group for the chatbot (mean 4.0, SD 0.82) and also the ClaimIt (mean 3.92, SD 0.76) app.

The preterm group felt that the length of interactions was between “long” to “neutral” (mean 2.92, SD 1.19; 1=too long, 5=easily manageable), while the term group felt that the length of interactions was between “manageable” and “easily manageable” (mean 4.31, SD 0.48). Furthermore, 46% (6/13) of the preterm parents and 23% (3/13) of the term parents experienced technical issues when using the chatbot.

Overall, participants were not worried about sharing their information (mean 4.04, SD 1.08; 1=very worried, 5=not at all worried) and were likely to use the chatbot again (mean 3.35, SD 0.75; 1=not at all likely, 5=very likely). Parents in both the term and preterm groups were generally not worried about data sharing and reported between “neutral” and “likely to use” chatbot technologies again to provide input on similar topics.

### Responses to Closed-Ended Questions on Stress, Sleep, and Feeding

Conversations from the 45 enrolled parents were analyzed. Parents with preterm infants reported emotional stress more frequently compared to parents with term infants (33 vs 24 occasions). Parents with term infants reported physical stress more frequently compared to parents with preterm infants (30 vs 10 occasions). When the cause of stress was not directly linked to their infants, parents with term infants reported stressors on more occasions (27 vs 18 occasions for the preterm group). Common stressors experienced by both preterm and term parents were breastfeeding, work, and relationships. Only parents of term infants reported breast-related issues (7 occasions).

**Figure 3:**
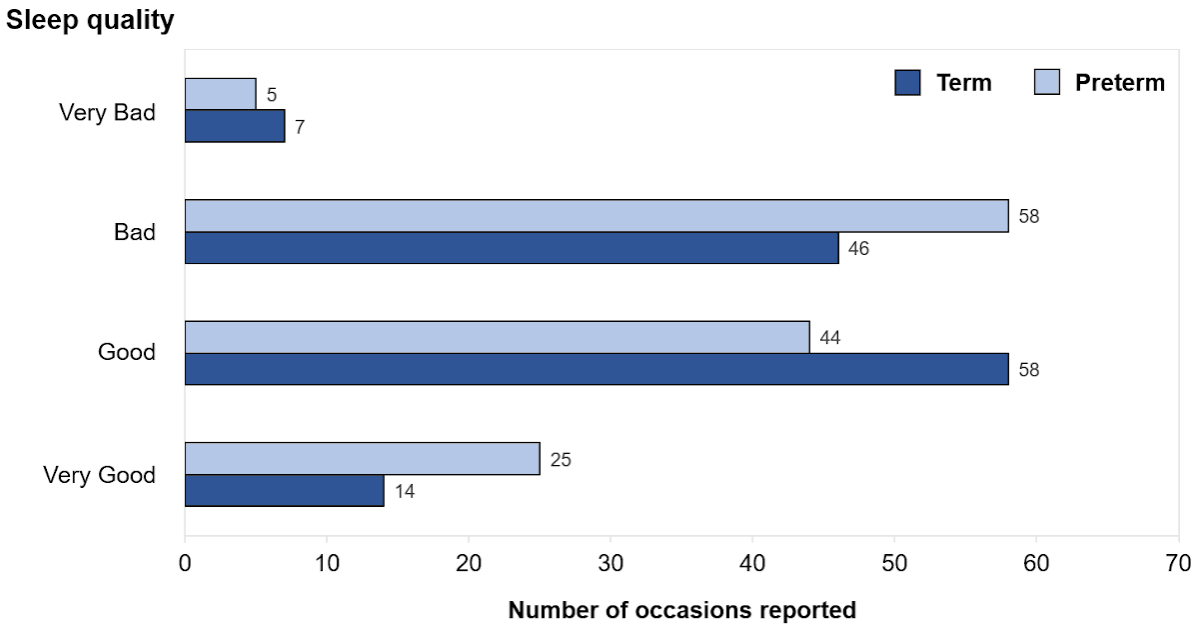
Rating of overall sleep quality by term and preterm group parents.

In general, parents perceived their sleep quality to be satisfactory although the preterm group reported good sleep slightly less frequently than did the term group (Figure 3). In terms of total sleep hours per day, preterm parents reported an average of 5.8 hours, while term parents reported an average of 6.1 hours.

Among parents who gave their infants breast milk, the most commonly reported feeding frequency was 8 to 11 times per day. This was true for both the preterm and term group. Among parents who gave their infants infant formula, the most commonly reported feeding frequency was 4 to 7 times per day in both the preterm and term groups. Feeding problems, such as irregular feeding, were more frequently reported by preterm group parents than by the term group parents (8 vs 2 occasions, respectively).

### Knowledge Inferred From Semantic Analysis of Chatbot Conversations

Open-ended responses to the conversation scripts from the 45 enrolled participants were used for the semantic analysis. Due to the limited length of the raw conversations, conversations belonging to the same participant were merged into a single conversation. This resulted in 39 conversations for the stress domain (17 term, 22 preterm) with an average conversation length of 27.4 words, 39 conversations for the sleep domain (17 term, 22 preterm) with an average conversation length of 28.5 words, and 40 conversations for the feeding domain (18 term, 22 preterm) with an average conversation length of 16.9 words (Table 1).

For the stress and sleep domains, in each LDA-derived model, the top 3 most representative words for each topic were found to be consistent across the 10 learning runs performed. For the feeding domain, topic composition across the 10 learning runs was characterized by a high degree of variability; that is, the top 3 most representative words of each topic varied across learning runs. Thus, an optimal and reproducible set of topics could not be learned from the conversations in the feeding domain. This could be due to the shorter length of feeding conversations compared with conversations from the stress and sleep domains.

**Figure 4:**
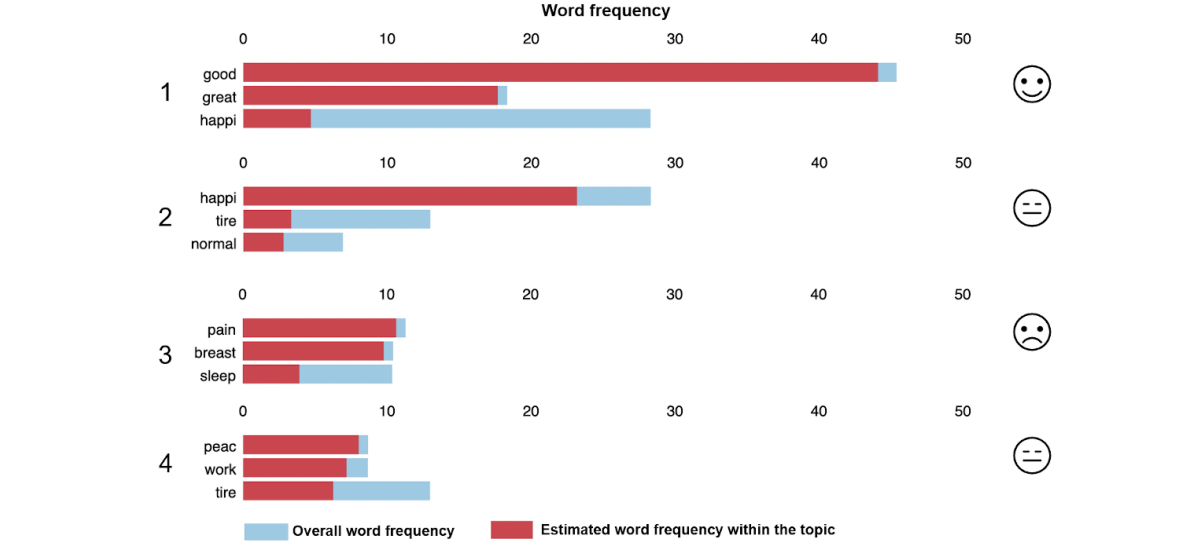
Three most representative words for each topic learned from conversations in the stress domain.

**Figure 5:**
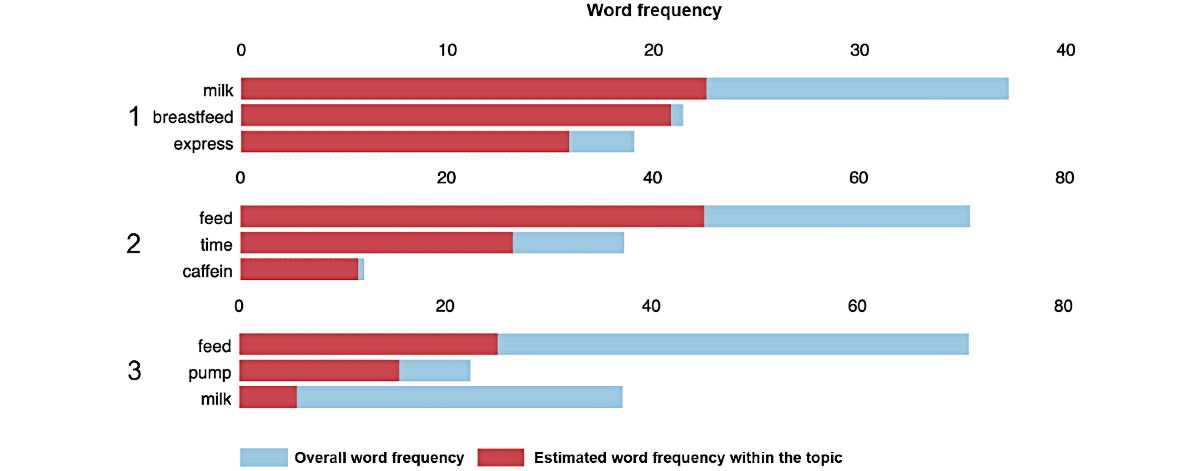
Three most representative words for each topic learned from conversations in the sleep domain.

For all 3 domains, models with 3 or 4 semantic topics were identified by human experts as being the most interpretable. The semantic topics for the stress (4 topics) and sleep (3 topics) domains inferred using the LDA topic modeling are shown in Figures 4 and 5, respectively. Only the top 3 most representative words for each topic are shown.

#### Stress Domain

In Figure 4, topic 1 appears to be linked to positive emotions and less stressful situations. Both topics 2 and 4 reflect “mixed feelings” of moderate well-being coupled with tiredness, whereas topic 3 appears to be associated with feelings of physical discomfort.

**Figure 6:**
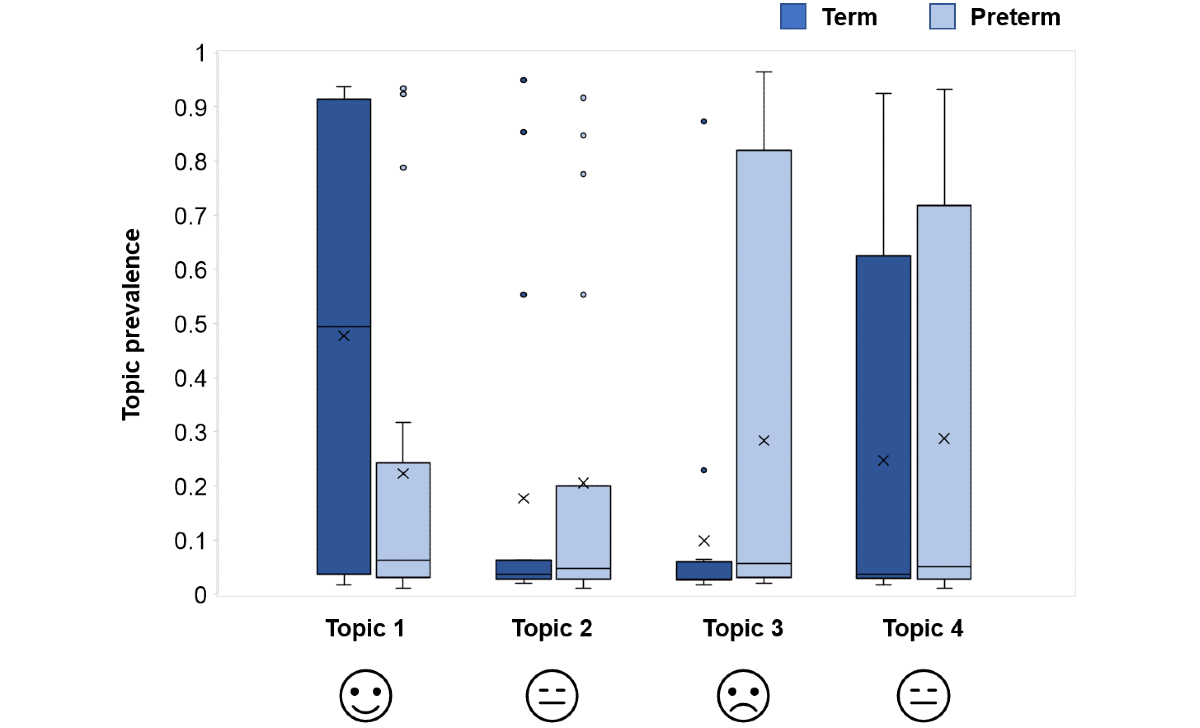
Topic prevalence in stress domain conversations from term and preterm group parents.

When the distribution of conversations over the 4 topics was calculated for each group (Figure 6), topics associated with opposite feelings (topic 1 and topic 3) exhibited dissimilar patterns for the term and preterm parents: topic 1 (positive) was the most prevalent topic in conversations of term parents, whereas preterm parents used words associated with this topic less frequently in their conversations. On the other hand, topic 3 (physical discomfort) appeared less frequently in conversations from term parents, whereas preterm parents made much more use of words belonging to this topic. The frequent occurrence of representative words for the “discomfort”-related topic (“pain,” “breast,” and “sleep”) in conversations from preterm group parents suggests this group experienced a higher degree of physical stress and discomfort.

#### Sleep Domain

Within the sleep domain, topic 1 appeared to be linked to breastfeeding, topic 2 to feeding in generic terms, and topic 3 to feeding using a milk pump (Figure 5). This showed that when parents were asked to comment on their sleep, their responses revolved around some aspects of feeding, suggesting that feeding might be interfering with parents’ ability to get an adequate amount of sleep or rest. When the distribution of conversations over the 3 topics (ie, the prevalence of the topics for each conversation) was calculated by group, term and preterm parents did not exhibit different semantic patterns in their conversations unlike those seen for the stress domain.

#### Feeding Domain

For this domain, the average conversation length was shorter than for the other 2 domains, resulting in a smaller feeding conversation data set. As a result, an optimal and reproducible set of topics could not be learned for the feeding domain. It is nonetheless interesting to note that feeding-related words and topics dominated the content of conversations collected for a different domain, sleep (Figure 5), suggesting close interactions between these 2 domains as perceived by parents in caring for their infants.

## Discussion

### Principal Findings

This study collected real-life, in-home data on parental stress, sleep, and infant feeding from parents of preterm and term infants using a chatbot. Participants who completed the study were satisfied with their online interactions with the chatbot and found the chatbot easy to use. Importantly, they were not worried about sharing such information through an interactive tool and were willing to use the chatbot to provide input on similar topics in the future. This finding helps to validate the use of chatbots on mobile devices as a convenient and accessible means of supporting parents of newborn infants and collecting data on topics that are important for the health and well-being of both infants and parents.

For the stress domain, the top conversation topic extracted from the semantic analysis showed strong positive emotions among parents with term infants. The other topics captured mixed feelings of moderate well-being and being tired, as well as general discomfort. Parents with preterm infants were more likely to express experiences of physical discomfort and tiredness through representative topic words like “pain,” “breast,” and “sleep.” The semantic analysis thus revealed a state of high physical stress in parents of preterm infants. In addition, they also reported emotional stress more frequently compared with term group parents. Similar experiences have been reported in earlier studies [2,3], especially in cases where the preterm infant was admitted to the ICU [5]. In our study, parents with term infants expressed positive emotions more frequently than did those in the preterm group. However, they were not spared the stress of caring for their infants, reporting physical stress on more occasions than the preterm group. With the addition of a new member to the family, noninfant-related stressors involving work and relationships were reported by both preterm and term group parents in this study.

An interesting insight from our semantic analysis of chatbot conversations on sleep was that the 3 most frequent topics of conversation for all parents (both the term and preterm groups) were related to feeding. This observation implies that parents intuitively linked feeding activities with their inability to have adequate rest. This could be explained by the need to feed their infants at regular intervals over the day and night. Indeed, the most commonly reported frequency of feeding was 8 to 11 times per day for breast milk and 4 to 7 times per day for infant formula. The close links between feeding and sleep revealed by semantic analysis adds another dimension to the closed-ended responses on sleep. Although both groups reported satisfactory sleep quality overall, preterm group parents reported good sleep quality slightly less frequently. Preterm group parents also reported feeding problems, such as irregular feeding, on more occasions than did term group parents.

### Limitations and Future Work

A total of 11 out of 45 enrolled participants (24%) were withdrawn from the study due to noncompliance (failing to complete the required number of chatbot interactions). For some participants, there were delays (up to 29 days) between enrolment and their first interaction with the chatbot. These delays could possibly be due to the stress experienced by parents and additional responsibilities of caring for a newborn at home after discharge. Although reminder notifications were sent on day 1 of each week, the next notification was only triggered on day 4 if the participant had not started a chat by that point. The high rates of noncompliance could be an indication of limited usability; for this reason, results for the usability questionnaire (answered by completers only) are presented descriptively and without attempting to perform statistical testing. Implementation of earlier and more frequent reminder notifications may improve participant compliance with chatbot interactions. Manual reminders via phone and external messaging platforms (WhatsApp and email) were implemented during the study to improve compliance and were well received. These reminders could be implemented in future work, along with further optimization of the technical performance of the mobile app and chatbot, to improve overall user experience and engagement in providing real-time data.

There were variations in word patterns believed to convey similar constructs that could pose some problems for completely unsupervised analysis. For example, in the stress and sleep scripts, participants were asked about how they were feeling and gave answers such as “good,” “not bad,” “doing well,” “god,” and “hood”. Intuitively, “good,” “not bad,” and “doing well” could be interpreted as saying that the person who responded felt “good,” However, without appropriate manual preprocessing, words such as “god” and “hood” might not be appropriately handled by the LDA algorithm. The conversation length was increased by merging multiple conversations to improve the efficiency of the LDA algorithm as discussed earlier. For the feeding domain, the average merged conversation length (16.9 words) was much shorter than for the other 2 domains (27-28 words). This resulted in a smaller feeding conversation data set and may explain why a reproducible set of topics could not be learned for this domain. Future studies should seek to validate the findings of this exploratory work with larger conversation data sets both in terms of the number or length of conversations and the number of participants. Additional topic modeling methods for short-text data could also be explored to improve handling of short or variable conversation length.

Although the 3 chatbot scripts (stress, sleep, and feeding) collected a large breadth of information, the depth of information was limited. The scripts explored the immediate concerns of parents and their high-level daily activities, but further studies are required to gain deeper insights. Future work could expand the scope of the chatbot to examine conversation topic patterns associated with other infant or family characteristics such as single or multiple births, parental age or age group, number of primary caregivers, or differences between first-time parents and those with more than one child. If data from different geographical regions can be collected, it may also be of interest to the explore similarities and differences among parents in different regions.

Our study shows that the application of machine learning to open-ended conversations elicited by a chatbot can provide additional insights beyond those provided by closed-ended questionnaire responses or descriptive statistics. Appropriately guided by human expert interpretation, unsupervised classification approaches such as LDA can reveal links or topics of interest within conversation data that may not have been anticipated. In addition, it has been suggested that conversational agents such as chatbots also help fulfil other emotional needs [10]. In our context, conversing with a chatbot could help parents overcome feelings of isolation, cope with negative feelings and obtain encouragement, and, at the same time, refine the process of communication on the daily issues they are struggling with.

### Conclusions

In this study, a chatbot was successfully used to collect real-time, open-ended conversation data on parental stress, sleep, and infant feeding. Using machine learning, our analysis of semantic patterns revealed differences between preterm and term group parents in conversation topic prevalence, notably for the stress domain. Positive emotions were more often expressed by parents with term infants, whereas parents with preterm infants more frequently expressed feelings of discomfort and tiredness, suggesting they were experiencing higher levels of stress. Topics involving infant feeding dominated the content of sleep domain conversations. Taken together with the results for self-reported sleep quality and feeding problems, these links between sleep and infant feeding suggest that preterm parents could have been more affected by poorer sleep related to frequent feeding or feeding problems. Overall, there was positive feedback from parents who completed the study on the usability experience of the chatbot as a tool to express their thoughts and concerns.

## Appendix

Multimedia Appendix 1 Chatbot scripts for stress, sleep, and feeding.

Multimedia Appendix 2 Usability eQuestionnaire for the chatbot and ClaimIt app.

Multimedia Appendix 3 Preprocessing of conversations to extract corpus for the latent Dirichlet allocation.

## Abbreviations

eQuestionnaire: electronic questionnaire
ICU: intensive care unit
LDA: latent Dirichlet allocation
LSA/LSI: latent semantic analysis/indexing
NMF: nonnegative matrix factorization
pLSA: probabilistic latent semantic analysis
