# Supplementary material for: A Chatbot to Engage Parents of Preterm and Term Infants on Parental Stress, Parental Sleep, and Infant Feeding: Usability and Feasibility Study
Source: JMIR Pediatr Parent. 2021 Oct 26;4(4):e30169. doi: 10.2196/30169 (PMC8579217; doi:10.2196/30169)
Supplement: Multimedia Appendix 1 [file pediatrics_v4i4e30169_app1.docx]

## Appendix 1: Chatbot scripts for stress, sleep and feeding

**Stress**

| **How are you feeling?** | | | | | | | | | | | | | | | | | | | | | |
| --- | --- | --- | --- | --- | --- | --- | --- | --- | --- | --- | --- | --- | --- | --- | --- | --- | --- | --- | --- | --- | --- |
| *[Collect open ended response]* | | | | | | | | | | | | | | | | | | | | | |
| **Have you felt stressed in the last few days?** | | | | | | | | | | | | | | | | | | | | | |
| No | | Yes | | | | | | | | | | | | | | | | | | | |
| **That’s great to hear! What do you think best describes your overall mood in the last day?** | | **Sorry to hear that! How often did you feel stressed? Occasionally? Regularly? Almost always?** | | | | | | | | | | | | | | | | | | | |
|  |  | Occasionally throughout the day | | | | | Regularly throughout the day | | | | | | | | | | | Almost always | | | |
|  |  | **I’m sorry to hear that! What do you think best describes the cause of your stress?** | | | | | | | | | | | | | | | | | | | |
|  |  | My baby | | | | | | | | | | | | | | | | My relationship | | Lack of sleep | Something else |
|  |  | **Again, I am sorry to hear that. Is there something in particular that is stressful?** | | | | | | | | | | | | | | | | **What in particular is stressful?** *[Collect open ended response]* | | | |
|  |  | My baby’s growth | My baby’s feeding habits | | | My baby is crying a lot | | My baby’s sleeping patterns | | | My baby is sick | | | | | Other | |  |  |  |  |
|  |  | **What is concerning you about their growth?** | **What is concerning about their feeding habits?** | | | **What is concerning about how much they are crying?** | | **What is most concerning?** | | | **Please describe the symptoms or conditions your baby has.** | | | | | **Please specify if there is something else about your baby that is stressing you out.** | |  |  |  |  |
|  |  | *[Collect open ended response]* | | | | | | | | | | | | | | | |  |  |  |  |
|  |  | **That is understandable. Is there anything else that is stressful for you?** | | | | | | | | | | | | | | | | | | | |
| *[Collect open ended response]* | | Yes | | | | | | | | | | | No | | | | | | | | |
|  |  | *[Return to question above]* | | | | | | | | | | |  |  |  |  |  |  |  |  |  |
|  |  | **I’m glad to hear that! Are your stressors affecting you physically, emotionally, or both?** | | | | | | | | | | | | | | | | | | | |
|  |  | Emotionally | | | | | | | | Physically | | | | | | | | | Both | | |
|  |  | **How does your stress affect you emotionally?** | | | | | | | | **How does your stress affect you physically?** | | | | | | | | | *[If both, ask both physical and emotional questions]* | | |
|  |  | Provide options: I feel irritable – scared – worried | | Other | | | | | | Provide options: I feel jittery – dizzy – sweaty – sick to my stomach | | | | | Other | | | |  |  |  |
|  |  |  |  | **Can you tell me how it affects you emotionally?** | | | | | |  |  |  |  |  | **Can you tell me how it affects you physically?** | | | |  |  |  |
|  |  |  |  | *[Collect open ended response]* | | | | | |  |  |  |  |  | *[Collect open ended response]* | | | |  |  |  |
|  |  | **Thanks for sharing that, and I’m sorry you’re affected by stress like this. Do you have an opportunity talk to anyone about how your stress affects you?** | | | | | | | | | | | | | | | | | | | |
| **Thanks for sharing that! Is there any type of support you think would be helpful in general?** | | Yes | | | | | | | | | | | | | | | | No | | | |
|  |  | **That’s great! Who do you talk to about it?** | | | | | | | | | | | | | | | |  |  |  |  |
|  |  | Friend | | | Family member | | | | Partner | | | | | Other | | | | **Well I’m glad we could chat about this! If you’re ever feeling overwhelmed, please reach out to your healthcare provider. They may be able to provide helpful solutions to you. Do you think there is there anything that could be done to help you feel less stressed?** | | | |
|  |  |  |  |  |  |  |  |  |  |  |  |  |  | *[Collect open ended response]* | | | |  |  |  |  |
|  |  | **I’m glad to hear you have someone to talk to about your stress! If you’re ever feeling overwhelmed, please reach out to your healthcare provider. They may be able to provide helpful solutions to you. Other than talking to someone, do you think there is there anything that could be done to help you feel less stressed?** | | | | | | | | | | | | | | | |  |  |  |  |
|  |  | Yes | | | | | | No | | | | | | | | | I don’t know | | | | |
|  |  | **What type of support would make you feel better?** *[Offer all options]* | | | | | | **I’m sorry to hear that. I have a few suggestions for you to think about!** | | | | | | | | | | | | | |
| No | Yes |  |  |  |  |  |  |  |  |  |  |  |  |  |  |  |  |  |  |  |  |
| **Thank you for chatting with me today!** | **Would you like some more information about preterm babies?** | | | | | | | | | | | **Would you like someone to talk to on the phone?** | | | | | | | | | |
|  | **This is a great resource for more information about preterm babies:**  [**http://www.bliss.org.uk**](http://www.bliss.org.uk) | | | | | | | | | | | **Here are a couple of links, so you can pick the one that best one for you!**  [**https://www.healthhub.sg/a-z/support-groups-and-others/20/call-on-these-when-you-need-help**](https://www.healthhub.sg/a-z/support-groups-and-others/20/call-on-these-when-you-need-help)  [**https://www.dumex.com.sg/careline**](https://www.dumex.com.sg/careline) | | | | | | | | | |
|  | **I hope you find these helpful! Just as a reminder, if you are feeling too stressed or need any more support than we are able to provide, contact your healthcare provider. Thank you for chatting with me today.** | | | | | | | | | | | | | | | | | | | | |

**Sleep**

| **How did you sleep last night?** | | | | | | | | | | | | | | | | | | | | | | | | | | |
| --- | --- | --- | --- | --- | --- | --- | --- | --- | --- | --- | --- | --- | --- | --- | --- | --- | --- | --- | --- | --- | --- | --- | --- | --- | --- | --- |
| Good | | | | | | | | | | | | | | Not well | | | | | | | | | | | | |
| **That’s great! Is your baby home with you or still at the hospital?** | | | | | | | | | | | | | | **I’m sorry to hear that. Is your baby home with you or still at the hospital?** | | | | | | | | | | | | |
| My baby is at home. | | | | | | | My baby is still at the hospital. | | | | | | | My baby is at home. | | | | | | | | | | My baby is still at the hospital. | | |
| **Thanks for sharing that. How many total hours of sleep did you get last night?**  *[Collect open ended numerical response and filter into below categories]* | | | | | | | | | | | | | | **Thanks for sharing that. How many total hours of sleep did you get last night?**  *[Collect open ended numerical response and filter into below categories]* | | | | | | | | | | | | |
| <7 hours | | | | ≥7 hours | | | | | I don’t know | | | | | <7 hours | | | | ≥7 hours | | | | | | | I don’t know | |
| **I’m glad you still feel well-rested. Did anything affect your sleep?** | | | | **That is a good amount of sleep! Did anything bother you about your sleep?** | | | | | **That’s ok, I only need an estimate.** | | | | | **What do you think affected your sleep?** | | | | **That is a good amount of sleep. Why do you feel like you didn’t sleep well? Did anything bother you about your sleep?** | | | | | | | **That’s ok, I only need an estimate.** | |
|  |  |  |  |  |  |  |  |  | *[Collect open ended numerical response]* | | | | |  |  |  |  |  |  |  |  |  |  |  | *[Collect open ended numerical response]* | |
|  |  |  |  |  |  |  |  |  | **Please share what may have affected your sleep last night.** | | | | |  |  |  |  |  |  |  |  |  |  |  | **Please share what may have affected your sleep last night.** | |
| *[All questions lead to these responses]* | | | | | | | | | | | | | | | | | | | | | | | | | | |
| I went to bed too late. | | | | | | | | | | | I woke up during the night. | | | | | | I woke up too early. | | | | | | | | Other | Nothing affected my sleep.  *[Skip to question below]* |
|  |  |  |  |  |  |  |  |  |  |  | **Sorry to hear that, do you remember how often you woke up?** *[Collect open ended response]* | | | | | |  |  |  |  |  |  |  |  |  |  |
| **Sorry to hear that, what in particular kept you awake?** | | | | | | | | | | | **What would you say woke you up?** | | | | | | | | | | | | | | **What do you feel affected your sleep?** |  |
| I couldn’t get to sleep | | | My baby | | | Other | | | | | I had to go to the bathroom | | My baby | Physical discomfort | | | | | I had a lot on my mind | | | Other | | |  |  |
| **Did it take you more than 30 minutes to get to sleep?** | | | **Please share what happened with your baby that kept you up.** | | | **Please share what kept you awake.** | | | | |  |  | **Please share what happened with your baby that kept you up.** | **Please share what made you feel physically uncomfortable (i.e. too hot, too cold, pain, etc.)** | | | | | **Please share what was on your mind.** | | | **Please share what kept you awake.** | | | *[Collect open ended response]* |  |
| No | Yes | |  |  |  |  |  |  |  |  |  |  | *[Collect open ended response]* | | | | | | | | | | | |  |  |
|  | **How come?** | |  |  |  |  |  |  |  |  | **Was there any other reason you may have been awoken?** | | | | | | | | | | | | | | |  |
|  | *[Collect open ended response]* | | | | | | | | | | Yes  *[Return to “****What would you say woke you up****?” above]* | | | | | | | | | No | | | | | |  |
| **Did anything else affect your sleep?** | | | | | | | | | | | | | | | | | | | | | | | | | |  |
| Yes | | | | | | | | | | | | | | | | | | | | | No | | | | |  |
| **What else affected your sleep?** *[Return to* ***“What do you think affected your sleep?”*** *responses above]* | | | | | | | | | | | | | | | | | | | | |  |  |  |  |  |  |
| **On a scale of 1 to 4 (1 being Very Good and 4 being Very Bad), how would you rate your quality of sleep last night?** | | | | | | | | | | | | | | | | | | | | | | | | | | |
| *[Collect open ended response]* | | | | | | | | | | | | | | | | | | | | | | | | | | |
| **Thanks for sharing that. How did you feel when you woke up this morning?** | | | | | | | | | | | | | | | | | | | | | | | | | | |
| Tired | | | | | | | | Well-rested | | | | | | | Other | | | | | | | | | | | |
|  |  |  |  |  |  |  |  |  |  |  |  |  |  |  | **How would you describe how you felt when you woke up this morning?** | | | | | | | | | | | |
|  |  |  |  |  |  |  |  |  |  |  |  |  |  |  | *[Collect open ended response]* | | | | | | | | | | | |
| **Did you still feel tired in the afternoon?** | | | | | | | | **That’s great! Did you feel tired in the afternoon?** | | | | | | | **Did you feel tired in the afternoon?** | | | | | | | | | | | |
| *[All questions lead to these responses]* | | | | | | | | | | | | | | | | | | | | | | | | | | |
| Yes | | | | | | | | | | | | | | | | | | | | | | | No | | | |
| **Did you take a nap or manage being tired in any other way?** | | | | | | | | | | | | | | | | | | | | | | | **I’m glad to hear that! Would you like some additional information on improving sleep quality overall?** | | | |
| Nap | | Caffeine | | | Medication | | | | | | | Other *[Collect open ended response]* | | | | No | | | | | | |  |  |  |  |
|  |  |  |  |  | **What medication did you take?** | | | | | | |  |  |  |  |  |  |  |  |  |  |  |  |  |  |  |
|  |  |  |  |  | *[Collect open ended response]* | | | | | | |  |  |  |  |  |  |  |  |  |  |  |  |  |  |  |
| **Did this help?** | | | | | | | | | | | | | | | |  |  |  |  |  |  |  |  |  |  |  |
| Yes | | | | | | | | | | No | | | | | |  |  |  |  |  |  |  |  |  |  |  |
| **I’m glad to hear that you’re feeling less tired! Would you like some additional information on improving sleep quality overall?** | | | | | | | | | | **I’m sorry to hear that. Would you find it helpful if we shared some additional information on improving sleep quality overall?** | | | | | | | | | | | | |  |  |  |  |
| Yes | | | | | | | | | | | | | | No | | | | | | | | | | | | |
| **Great! This link has a lot of information about improving sleep and sleep quality:** [**http://www.healthhub.sg/live-healthy/510/sleep**](http://www.healthhub.sg/live-healthy/510/sleep) | | | | | | | | | | | | | |  |  |  |  |  |  |  |  |  |  |  |  |  |
| **Thanks for chatting with me today!** | | | | | | | | | | | | | | | | | | | | | | | | | | |

**Feeding**

| **Over the last 24 hours what have you primarily been feeding your baby?** | | | | | | | | | | | | | | | | | | | | | | | | | | | | | |
| --- | --- | --- | --- | --- | --- | --- | --- | --- | --- | --- | --- | --- | --- | --- | --- | --- | --- | --- | --- | --- | --- | --- | --- | --- | --- | --- | --- | --- | --- |
| Breastmilk | | | | | | | | | | | | Infant formula | | | | | | | | | | Other | | | | | | | |
| **Did you feed your baby using a bottle or via direct breastfeeding?** | | | | | | | | | | | | **How many times did you give your baby formula in that time?** | | | | | | | | | | **What have you fed your baby in that time?** | | | | | | | |
| Direct breastfeeding | | | Bottle | | | | | Both | | | | 0-3 | 4-7 | 8-11 | 12+ | | I don’t know | | | | | Milk alternative or beverage | | | | Weaning food | | | |
| **That’s great! How many times did you breastfeed in that time?** | | | **Great! I understand you gave your breastmilk with a bottle. Please let me know how many times.** | | | | | **How many times did you breastfeed or give breastmilk through a bottle in that time?** | | | |  |  |  |  |  | **That’s okay- an estimate is all I need.** | | | | |  |  |  |  |  |  |  |  |
| 0-3 | 4-7 | 8-11 | | 12+ | | I don’t know | | | | | |  |  |  |  |  | *[Provide values]* | | | | |  |  |  |  |  |  |  |  |
|  |  |  |  |  |  | **That’s okay- please give me an estimate.** | | | | | | **Please tell me the brand and product name of the formula used.** | | | | | | | | | | **That’s great! Which milk alternative(s) or beverage(s) did your child have?** | | | | **OK, what type of weaning food did your child have?** | | | |
|  |  |  |  |  |  | *[Provide values]* | | | | | | *[Collect open ended response]* | | | | | | | | | |  |  |  |  |  |  |  |  |
| *[If answered* ***direct latching****, continue directly to response below]* | | | **When feeding with a bottle, how much milk did you give your baby?** | | | | | | | | |  |  |  |  |  |  |  |  |  |  | *[Collect open ended response]* | | | | *[Collect open ended response]* | | | |
|  |  |  | *[Collect open ended response]* | | | | | | | | |  |  |  |  |  |  |  |  |  |  | **Thank you for your response! How many times did you feed your baby milk alternative/weaning food in that time?** | | | | | | | |
|  |  |  | **Did your baby drink it all?** | | | | | | | | | **Great! How many bottles of formula did you give your baby in the last 24 hours?** | | | | | | | | | |  |  |  |  |  |  |  |  |
|  |  |  | Yes | | No | | | | | | | *[Collect open ended response]* | | | | | | | | | |  |  |  |  |  |  |  |  |
|  |  |  |  |  | **That’s ok. About how much did your baby drink?** | | | | | | | **Did your baby drink it all?** | | | | | | | | | | 0-3 | 4-7 | 8-11 | 12+ | | | I don’t know | |
|  |  |  |  |  |  |  |  |  |  |  |  | Yes | No | | | | | | | | |  |  |  |  |  |  | **No problem, you can give me an estimate.** | |
|  |  |  |  |  |  |  |  |  |  |  |  |  | **That’s ok. About how much did your baby drink?** | | | | | | | | |  |  |  |  |  |  |  |  |
|  |  |  |  |  | *[Collect open ended response]* | | | | | | |  | *[Collect open ended response]* | | | | | | | | |  |  |  |  |  |  | *[Provide values]* | |
| **Thanks for sharing that! Do you also feed your baby infant formula or a milk alternative?** | | | | | | | | | | | | **Thanks for sharing that! Do you also feed your baby breast milk or a milk alternative?** | | | | | | | | | | **Thanks for sharing that! Do you also feed your baby breast milk or formula?** | | | | | | | |
| Yes | | | | | | | | | | No | | Yes | | | | | | | No | | | Yes | | | | | | | No |
| **Please share whether your child had infant formula or a milk alternative in the last 24 hours?** *[return to first responses]* | | | | | | | | | |  |  | **Please share whether your child had breast milk or a milk alternative in the last 24 hours?** *[return to first responses]* | | | | | | |  |  |  | **Please share whether your child had breastmilk or infant formula in the last 24 hours?** *[return to first responses]* | | | | | | |  |
| **Were you happy with your baby’s feeding habits over the last 24 hours?** | | | | | | | | | | | | | | | | | | | | | | | | | | | | | |
| No | | | | | | | | | | | | | | | | | | | | | | | | | | | Yes | | |
| **Did you feel your baby consumed more or less than usual in the last 24 hours?** | | | | | | | | | | | | | | | | | | | | | | | | | | |  |  |  |
| Less than usual | | | | | | | | | | | | | More than usual | | | | | | | | | | | | | |  |  |  |
| **Why do you think your baby consumed less than usual?** | | | | | | | | | | | | | **Why do you think your baby consumed more than usual?** | | | | | | | | | | | | | |  |  |  |
| *Both questions lead to the responses below* | | | | | | | | | | | | | | | | | | | | | | | | | | |  |  |  |
| My baby is sick | | | | | | | | | My baby is teething | | | | I don’t know | | | | | | | Other | | | | | | |  |  |  |
| **I’m sorry to hear that. What symptoms or conditions does your baby have that make you worry they’re sick?** | | | | | | | | |  |  |  |  | **Are you worried about your baby’s change in feeding habits?** | | | | | | | *[Collect open ended response]* | | | | | | |  |  |  |
|  |  |  |  |  |  |  |  |  |  |  |  |  | Yes | | | | | No | |  |  |  |  |  |  |  |  |  |  |
| *[Collect open ended response]* | | | | | | | | |  |  |  |  | **I’m sorry to hear that. Can you describe your concerns?** | | | | |  |  |  |  |  |  |  |  |  |  |  |  |
|  |  |  |  |  |  |  |  |  |  |  |  |  | *[Collect open ended response]* | | | | |  |  |  |  |  |  |  |  |  |  |  |  |
| **A change in feeding habits is often nothing to worry about, but you can reach out your healthcare professional anytime you sense something is wrong. Have you done anything in response to your baby’s change in feeding habits?** | | | | | | | | | | | | | | | | | | | | | | | | | | |  |  |  |
| Yes | | | | | | | | | | | | | | | | | | | | | No, I observed my baby | | | | | |  |  |  |
| **Can you share with me what you did?** | | | | | | | | | | | | | | | | | | | | | **Did your baby’s feeding habits change at all? If so, how?** | | | | | |  |  |  |
| I spoke with the healthcare provider | | | | | | | I changed my baby’s diet | | | | | I used a home remedy | | | Other | | | | | |  |  |  |  |  |  |  |  |  |
| **That’s great! What did your healthcare provider suggest?** | | | | | | | **What did you do differently?** | | | | | **Which home remedy did you use? How did it help?** | | | **Which alternative action did you use to help with your baby’s appetite? Did your baby’s appetite improve?** | | | | | |  |  |  |  |  |  | **Do you have any concerns regarding your baby’s feeding habits?** | | |
| *[Collect open ended response]* | | | | | | | | | | | | | | | | | | | | | | | | | | |  |  |  |
| **Do you have any other concerns regarding your baby’s feeding habits?** | | | | | | | | | | | | | | | | | | | | | | | | | | |  |  |  |
| Yes | | | | | | | | | | | | | | | | | | | | | | No | | | | | | | |
| **What concerns do you have regarding your baby’s feeding habits?** | | | | | | | | | | | | | | | | | | | | | |  |  |  |  |  |  |  |  |
| *[Collect open ended response]* | | | | | | | | | | | | | | | | | | | | | |  |  |  |  |  |  |  |  |
| **Do you have any concerns regarding your baby’s growth?** | | | | | | | | | | | | | | | | | | | | | | | | | | | | | |
| Yes | | | | | | | | | | | | | | | | | | | | | | No | | | | | | | |
| **Can you share with me the concerns you are having to his/her growth?** | | | | | | | | | | | | | | | | | | | | | |  |  |  |  |  |  |  |  |
| *[Collect open ended response]* | | | | | | | | | | | | | | | | | | | | | |  |  |  |  |  |  |  |  |
| **Do you monitor the growth of your baby? If yes, how?** | | | | | | | | | | | | | | | | | | | | | | | | | | | | | |
| Growth Chart | | | | | | | | | | | At-home scale | | | | | Doctor visit | | | | | | | | | | | Other *[Collect open ended response]* | | |
| **If you ever have any concerns about your baby’s feeding habits or growth, please be sure to reach out to your healthcare provider. Thanks for chatting with me today!** | | | | | | | | | | | | | | | | | | | | | | | | | | | | | |
