# Supplementary material for: A Chatbot to Engage Parents of Preterm and Term Infants on Parental Stress, Parental Sleep, and Infant Feeding: Usability and Feasibility Study
Source: JMIR Pediatr Parent. 2021 Oct 26;4(4):e30169. doi: 10.2196/30169 (PMC8579217; doi:10.2196/30169)
Supplement: Multimedia Appendix 2 [file pediatrics_v4i4e30169_app2.docx]

## Appendix 2: Usability eQuestionnaire for chatbot and ClaimIt app

*The following is a questionnaire is for you to provide feedback on your interactions with the chatbot. Please pick the best answer.*

1. Please rate the study chatbot’s overall ease of use on a scale of 1 to 5:

(Very Difficult) 1 2 3 4 5 (Very Easy)

1. Please rate the ClaimIt application's overall ease of use on a scale of 1 to 5:

(Very Difficult) 1 2 3 4 5 (Very Easy)

1. How satisfied were you with your interactions with the study chatbot?

(Very Dissatisfied) 1 2 3 4 5 (Very Satisfied)

1. How satisfied were you with your interactions with ClaimIt?

(Very Dissatisfied) 1 2 3 4 5 (Very Satisfied)

1. Did the study chatbot malfunction?
   1. Yes (will be asked a follow-up question)
   2. No

*(If Yes)* How did the study chatbot malfunction? ____________ *[Open-ended question]*

1. Did ClaimIt malfunction?
   1. Yes (will be asked a follow-up question)
   2. No

*(If Yes)* How did ClaimIt malfunction? [Open-ended question]

1. How likely are you to consider using a chatbot application as an interactive tool to provide input on similar topics?

(Not at all Likely) 1 2 3 4 5 (Very Likely)

1. How would you rate the length of interactions you had with the study chatbot?

(Too Long) 1 2 3 4 5 (Easily Manageable)

1. What did you like the least about the study chatbot? ____________ *[Open-ended question]*
2. What did you like the most about the study chatbot? _____________ *[Open-ended question]*
3. Do you have any other comments about the study chatbot that you would like the investigators to know? __________________________________ *[Open-ended question]*
4. What did you like the least about the ClaimIt app? ____________ *[Open-ended question]*
5. What did you like the most about the ClaimIt app? _____________ *[Open-ended question]*
6. Do you have any other comments about the ClaimIt app that you would like the investigators to know? __________________________________ *[Open-ended question]*
7. How worried were you about sharing your data with the study chatbot?

(Not at all worried) 1 2 3 4 5 (Very worried)

1. How likely would you be to use a chatbot tool like this in real life to get access to information and reassurance if you could?

(Not at all likely) 1 2 3 4 5 (Very likely)

Why or why not? ____________ *[Open-ended question]*
