# Supplementary figures and images for: A Chatbot to Engage Parents of Preterm and Term Infants on Parental Stress, Parental Sleep, and Infant Feeding: Usability and Feasibility Study
Source: JMIR Pediatr Parent. 2021 Oct 26;4(4):e30169. doi: 10.2196/30169 (PMC8579217; doi:10.2196/30169)

## Appendix 3: Pre-processing of conversations to extract corpus for Latent Dirichlet Allocation


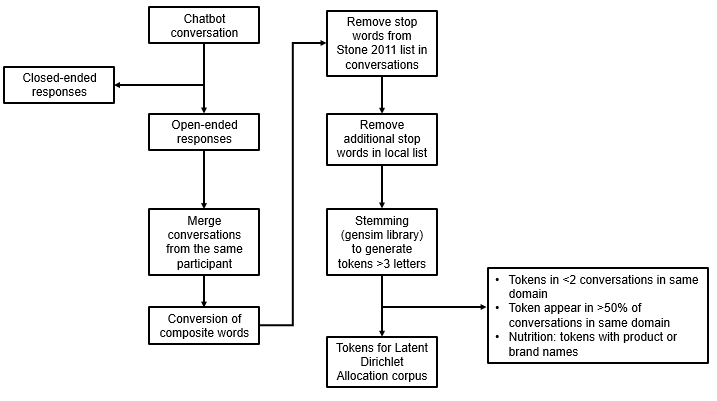

Supplement: Multimedia Appendix 3 [file pediatrics_v4i4e30169_app3.docx]
